# Residence of mice in metabolic cages reduces experimental kidney inflammation through stress-induced glucocorticoids

**DOI:** 10.1172/jci.insight.189794

**Published:** 2025-04-08

**Authors:** Junping Yin, Melanie Eichler, Clivia Lisowski, Jian Li, Sibylle von Vietinghoff, Natalio Garbi, Qi Mei, Anne-Kathrin Gellner, Christian Kurts

**Affiliations:** 1 Institute of Molecular Medicine and Experimental Immunology and; 2 Nephrology Section, Medical Clinic 1, University Hospital Bonn, Bonn, Germany.; 3 Department of Oncology, Tongji Hospital, Wuhan, China.; 4 Cancer Center, Shanxi Bethune Hospital, Taiyuan, China.; 5 Institute of Physiology II, Medical Faculty, University of Bonn, Bonn, Germany.

**Keywords:** Inflammation, Nephrology, Cellular immune response

## To the editor:

Metabolic cages are widely used to collect urine and feces from rodents in disease studies in various disciplines. These cages allow, for example, determining creatinine clearance and daily proteinuria, both of which are standard analytical endpoints in studies on experimental glomerulonephritis. Crescentic glomerulonephritis (cGN) is the most severe form of immune-mediated glomerulonephritis that rapidly progresses to terminal kidney failure unless immunosuppressive treatment is applied (1). A widely used animal model of this disease has uncovered important roles of dendritic cells, macrophages, Th1 and Th17 cells, and other immune cells. These mechanistic insights have facilitated the development of new therapies in patients with cGN (1–3).

**Figure 1:**
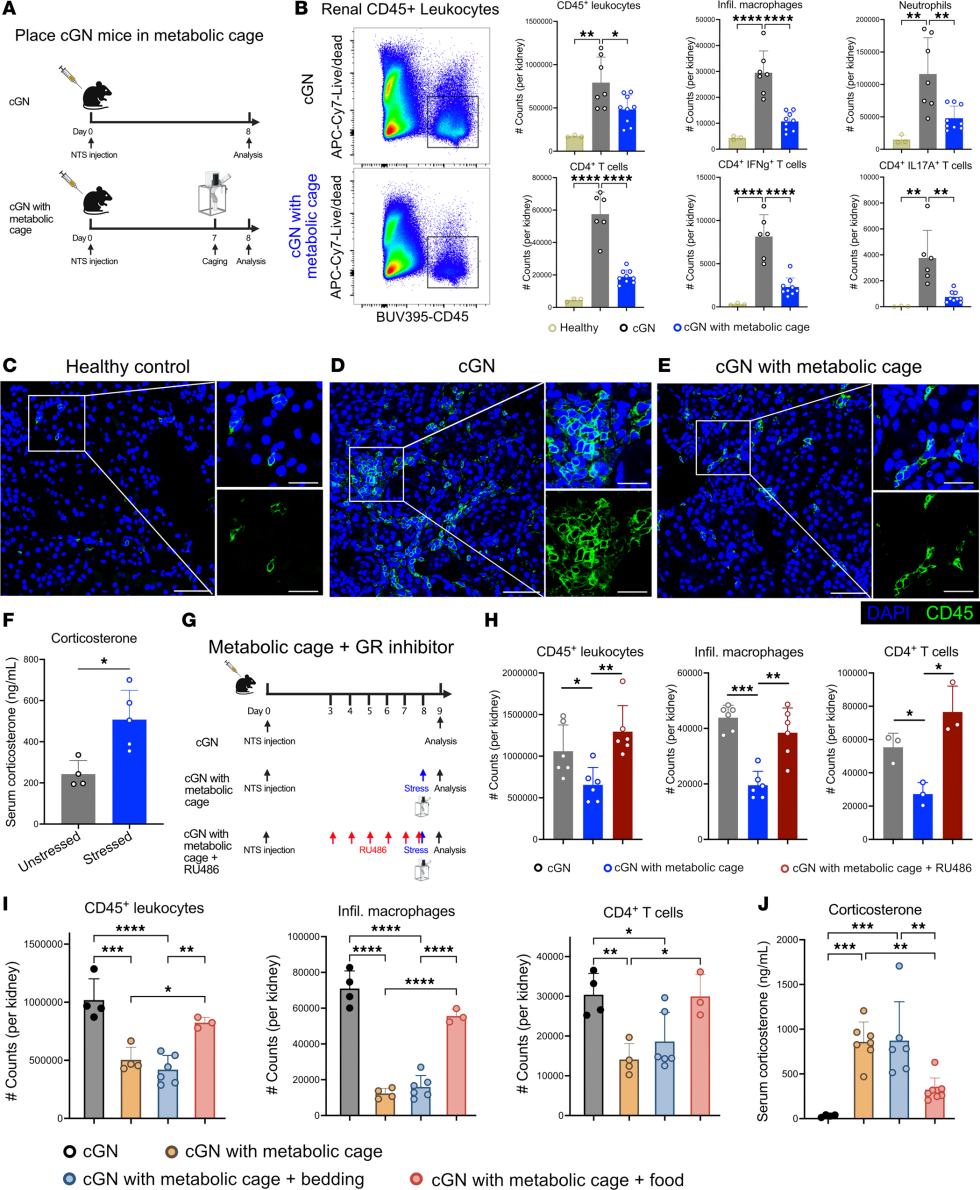
Metabolic cages suppress renal inflammation during experimental cGN. (**A**) Experimental plan. NTS, nephrotoxic serum. (**B**) Representative flow cytometry plot for renal CD45^+^ leukocytes from cGN mice in metabolic cages or not. Bar plots showing alterations of immune cells, infiltrating macrophages, neutrophils, CD4^+^ T cells, and Th1 and Th17 cells from healthy mice, cGN mice in metabolic cages (*n* = 9), or cGN mice not in metabolic cages (*n* = 7). (**C**–**E**) Representative confocal micrographs of kidney cryosections from healthy mice (**C**), cGN mice (**D**), and cGN mice in metabolic cages (**E**). Scale bar: 50 μm (left), 20 μm (right). (**F**) Corticosterone serum concentrations of unstressed (*n* = 4) and stressed (*n* = 5). (**G**) Experimental plan for determining the effect of the glucocorticoid receptor inhibitor RU486 on the progression of cGN. (**H**) Bar plots showing the renal infiltration of CD45^+^ leukocytes (*n* = 6), infiltrating macrophages (*n* = 6), and CD4^+^ T cells (*n* = 3). (**I**) Bar plots showing renal infiltration by immune cells (*n* = 4, 4, 6, 3 for groups from left to right). (**J**) Bar plots showing serum corticosterone levels. *n* = 4, 7, 6, 8 for groups from left to right. **B**, **H**, and **J** show data merged from 2 experiments; **F** and **I**, show representative data from 2 experiments. Data are presented as mean ± SD. Statistical analysis was done with 2 tailed Student’s *t* test or 1-way ANOVA. *P < 0.05, **P < 0.01, ***P < 0.001, ****P < 0.0001.

During our own studies in this model, we noted that the characteristic mononuclear immune cell infiltrate in the kidney tubulointerstitium was noticeably reduced in all mice from 3 of 5 experiments compared with all mice from the other 2 experiments. We eventually realized that, in those 3 experiments, mice had been placed for 16 hours overnight in metabolic cages to collect urine to determine daily proteinuria. We then performed a controlled experiment, where we placed one group of mice in metabolic cages but not another group, and compared intrarenal immune cell infiltration. Indeed, significantly fewer CD45^^+^^ leukocytes, including macrophages, neutrophils, and several subsets of CD4^^+^^ T cells, were present in kidneys of mice that had resided in metabolic cages, as detected by flow cytometry (Figure 1, A and B; Supplemental Figure 1, A and B; and Supplemental Methods; supplemental material available online with this article; https://doi.org/10.1172/jci.insight.189794DS1) and immunofluorescence microscopy (Figure 1, C–E). The crescent numbers were not significantly reduced (not shown). The immune cell infiltrate was still reduced 2 days after residence in metabolic cages, and a partial reduction was detectable after 4 days (Supplemental Figure 2, A–C).

We reasoned that keeping mice in a restrained space will stress the animals. Acute stress transiently activates the hypothalamic/pituitary/adrenal axis, which increases circulating glucocorticoid stress hormones, with potential suppressive effects on the immune system (2). Indeed, we noted that serum levels of corticosterone — the main rodent glucocorticoid, which is typically increased following acute restraint (4) — was higher after residence in metabolic cages (Figure 1F). Furthermore, in stressed mice with cGN, renal macrophages and CD4^+^ T cells expressed more glucocorticoid-target genes such as *Gilz* and *Sgk1* (Supplemental Figure 3). To establish causality, we treated nephritic mice with a glucocorticoid receptor inhibitor before placing them into metabolic cages (Figure 1G). Indeed, this restored intrarenal inflammation in stressed mice to numbers in nonstressed mice that had not been placed in metabolic cages (Figure 1H).

Placing mice in metabolic cages results in social deprivation, absence of bedding, and shelter and in food unavailability (Supplemental Figure 4, A and B). To determine which factors had stressed the mice, we placed either food or bedding and shelter into the metabolic cages (Supplemental Figure 4, A and B). Adding food alone restored nephritis, but adding bedding and shelter did not (Figure 1I and Supplemental Figure 4C), suggesting that food deprivation was the main factor that attenuated inflammation in experimental cGN. In support of this, corticosterone serum concentrations of mice in metabolic cages were no longer elevated when food was added, but they were elevated when bedding was provided without food (Figure 1J). Kidney function was not altered by removing bedding or food (Supplemental Figure 4D).

These findings demonstrate that a diagnostic procedure — here, the use of metabolic cages — can confound studies on glomerulonephritis. This likely will apply to other inflammatory disease models as well that involve stressful diagnostic procedures, given that glucocorticoids act systemically. Furthermore, our findings support the notion that fasting may affect immune responses (5). In support of this, caloric restriction has been shown to activate nutrient-sensing hypothalamic neurons, which stimulated the hypothalamic/pituitary/adrenal axis, resulting in antiinflammatory signals (6). Furthermore, diurnal rhythms, which prominently affect glucocorticoids, can alter the course of glomerulonephritis (3).

Our findings may help in explaining why animal studies are sometimes difficult to reproduce, even if inbred strains are used. Environmental differences between animal facilities, especially the local microbiome, are considered responsible. Our findings document that the individual stress experience of mice is another such difference. Therefore, placing only some mice into metabolic cages may cause systematic errors. Providing food can largely remedy this problem. This is usually not done because food fragments will contaminate the collected urine and perturb proteinuria measurements. If this parameter is of interest, alternative readouts for the intactness of the glomerular filter, such as the i.v. injection of fluorescent tracers, need to be used. Future studies may examine whether stress by social deprivation or altered social thermoregulation affect kidney inflammation as well.

## Supplementary Material

Supplemental data

Supporting data values

## Version 1 04/08/2025

In-Press Preview

## Version 2 05/08/2025

Electronic publication

## References

[1] Anders HJ (2023). Glomerulonephritis: immunopathogenesis and immunotherapy. Nat Rev Immunol.

[2] Jobin K (2020). A high-salt diet compromises antibacterial neutrophil responses through hormonal perturbation. Sci Transl Med.

[3] Mohandas R (2022). Circadian rhythms and renal pathophysiology. J Clin Invest.

[4] Ding JX (2021). Physical restraint mouse models to assess immune responses under stress with or without habituation. STAR Protoc.

[5] Kim BH (2021). Effects of intermittent fasting on the circulating levels and circadian rhythms of hormones. Endocrinol Metab (Seoul).

[6] Klima ML (2023). Anti-inflammatory effects of hunger are transmitted to the periphery via projection-specific AgRP circuits. Cell Rep.

