# Supplementary material for: Residence of mice in metabolic cages reduces experimental kidney inflammation through stress-induced glucocorticoids
Source: JCI Insight. 2025 Apr 8;10(9):e189794. doi: 10.1172/jci.insight.189794 (PMC12140023; doi:10.1172/jci.insight.189794)
Supplement: Supplemental data [file jciinsight-10-189794-s002.pdf]

Supplementary Methods

Mice

C57BL/6J male mice were purchased from Charles River and housed in the specific pathogen free (SPF) area of the central animal facility of University Clinic of Bonn. To induce crescentic glomerulonephritis (cGN), mice were intraperitoneally (i.p.) injected with nephrotoxic serum (NTS) against the glomerular basement membrane (2.5mg/g bodyweight). To collect the urine, mice were put individually in the metabolic cages (Tecniplast, Germany) overnight from 3pm to 7am. Mice were single-housed in transparent metabolic cages of about 200 cm^2 without food and bedding but with access to water ad libitum. Mice were sacrificed with CO^2 to collect organs and sera for analysis. Animal rooms had a light/dark cycle of 12/12h (6am-6pm). The inhibitor of glucocorticoid receptor (RU486, Sigma) was s.c. administered (1mg/mouse) from day 3 to day 8 after induction of cGN. Urinary albumin was measured by ELISA (Biomol, E99-134), urinary creatinine by standard laboratory protocols in the central laboratory of University Clinic of Bonn. Corticosterone serum concentrations were determined by ELISA (Enzo, ADI-901-097) according to manufacturer's manual.

Isolation and flow cytometric analysis of renal leukocytes

For flow cytometry of renal leukocytes, mice were sacrificed and perfused with cold PBS. Perfused kidneys were collected and digested with DNase and collagenase at 37 °C incubator, then filtered with 70µm cell strainers. Kidney single cell suspensions were stained with fluorochrome-conjugated antibodies (BUV395-anti-CD45 (BD, 564279), PerCP-Cy5.5-anti-CD11B (Biolegend, 101228), PE-anti-F4/80 (Biolegend, 123110), BV421-anti-CD11C (BD, 562782), AF700-anti-MHC II (Biolegend, 107622), FITC-anti-Ly6C (Biolegend, 128005), BV510-anti-Ly6G (Biolegend, 127633), AF700-anti-CD3 (Biolegend, 100216), PerCP-Cy5.5-anti-CD4 (Biolegend, 100434), BV510-anti-CD8 (Biolegend, 100752), APC-anti-IFNG (Biolegend, 505810), BV421-anti-IL17A (Biolegend, 506925)). For intracellular staining, cells were processed using the Foxp3 fix/perm kit following manufacturer's protocol. For staining the cytokines IL-17A and IFNg in T cells, single cell suspensions from kidneys were stimulated with PMA, ionomycin, Golgi-plug and Golgi-stop for 3 hours at the 37 °C CO2 incubator. Samples were measured by using BD LSRFortessa flow cytometer. Recorded data were analyzed in Flowjo 10 software.

Cell sorting

To sort renal neutrophils for analyzing the gene expression, $CD45^+$ leukocytes were enriched from kidney single cell suspensions by using CD45 MicroBeads (Miltenyi Biotec). Enriched $CD45^+$ leukocytes were used for sorting macrophages and $CD4^+$ T cells which were sorted in RNA lysis buffer with β-mercaptoethanol for RNA isolation.

Quantitative RTPCR

Total RNA from the kidney or sorted cells was isolated with the NucleoSpin Kit (Macharey-Nagel) in accordance with the manufacturer's protocol. RNA was then reverse transcribed with the High-Capacity cDNA Reverse Transcription Kit (Thermo Fisher). Real-time qPCR was performed using SYBR Green techniques (Life Technologies) with specific primers for *Gapdh (QT01658692, Qiagen), Gilz(QT01552005, Qiagen), Sgk1(*qMmuCED0045443*, BioRad)* using a LightCycler 480 II (Roche).

Histopathology and immunofluorescence

For immunofluorescence staining, murine kidneys were collected and fixed in PLP buffer overnight and followed by 30% sucrose in PBS for 24 hours at 4°. Afterwards, each kidney was embedded in the OCT and stored in -80 freezer and cut into 5 um sections on the slides. Cryo-sections (5 um) were rehydrated and blocked in 1% BSA solution for 1 h at room temperature. All blocked sections were then incubated at 4°C overnight with Alexa-Fluo 488 conjugated anti mouse CD45 antibodies (103122, Biolegend). In the next day, sections were counterstained with 4′,6-diamidino-2-phenylindole (DAPI, Sigma) and mounted with Fluoromount medium (Invitrogen). Fluorescence images were captured using Leica SP8 confocal microscope system. All immunofluorescence images were processed in Fiji software.

Statistical analysis

All plots and corresponding statistical analysis were performed in Graphpad Prism 10. The statistical significance of two individual groups was calculated by using Student's t test or Mann Whitney u test depending on the normality of distribution, while the significance for 3 or 4 groups was determined by using ANOVA test. A p value less than 0.05 was considered statistically significant.

Sex as a biological variable

Our study examined the male mice. It is unclear whether our findings apply to female mice.

Study approval

All animal studies had been approved by the local authority Landesamt für Natur, Umwelt und Verbraucherschutz of North Rhine-Westphalia, Germany.

Data availability

A single excel file containing values for all data points in the figures of this study is available in the Supporting Data Values file.

Acknowledgements

We acknowledge support by the Flow Cytometry Core Facility, Microscope Core Facility and the animal facilities of the Medical Faculty of Bonn University. This work was supported by the German Research Foundation (DFG) through SFB1192 No. 264599542, IRTG2168 No. 272482170, TRR237 No. 369799452, KFO329 No. 386793560, SFB1454 No. 432325352 and EXC2151 No. 390873048 and by a Gottfried Wilhelm Leibniz Award to CK.

Author contributions

J.Y. and C.K. conceptualized the study and wrote the paper. J.Y. and M.E. performed the experiments. C.K. acquired funding. All authors discussed the data and contributed to the final manuscript.

Supplementary Figures

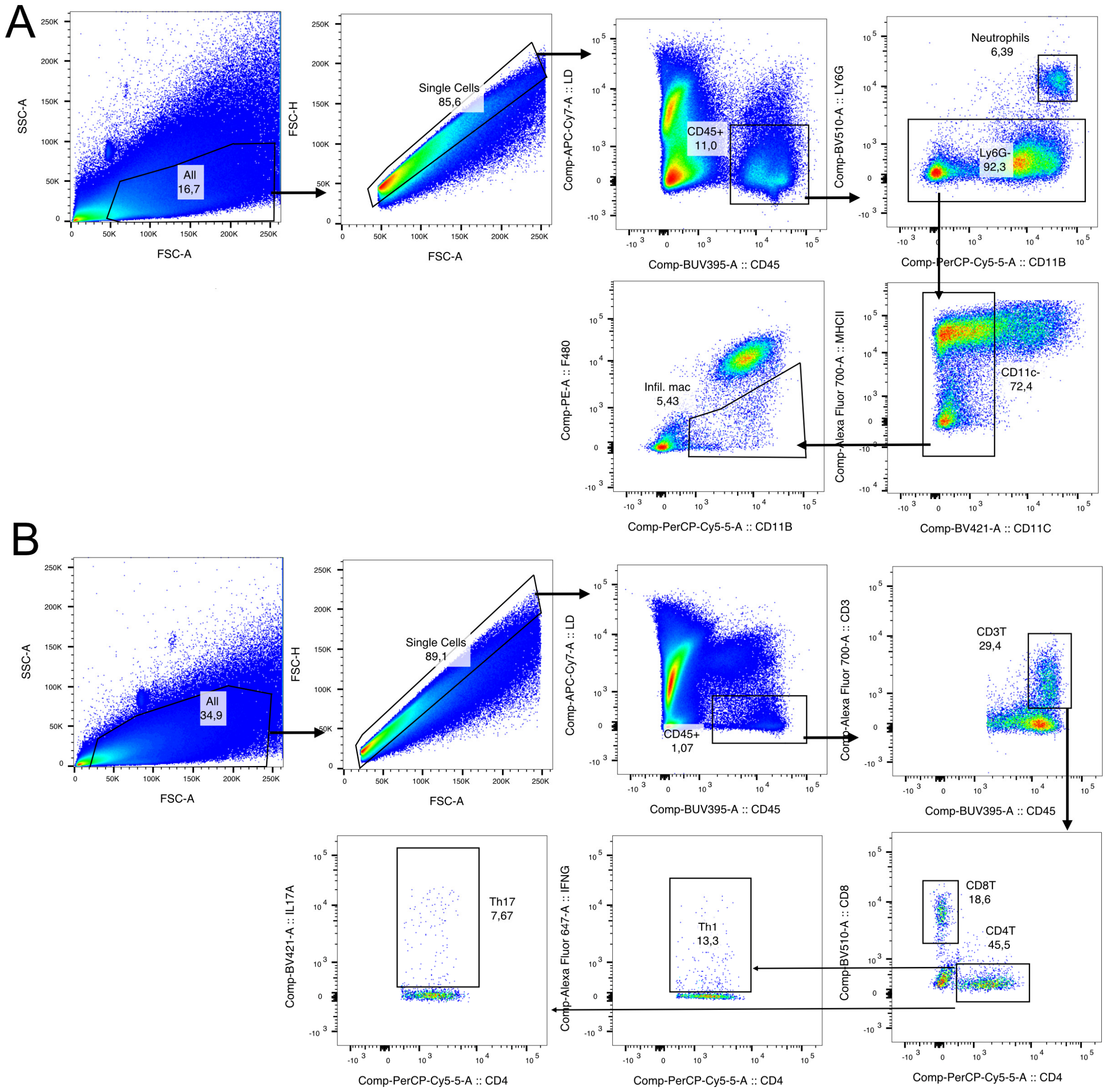

Supplemental Fig. 1. Flow cytometric analysis for the renal leukocytes. Gating strategy for renal $CD45^+$ leukocytes, neutrophils ($CD45^+Ly6G^+CD11b^+$) and infiltrated macrophages ($CD45^+Ly6G^-F4/80^{low/-}CD11b^+$) (**A**) and $CD4^+$ T cells, $IFNg^+$ Th1 cells and $IL17A^+$ Th17 cells (**B**).

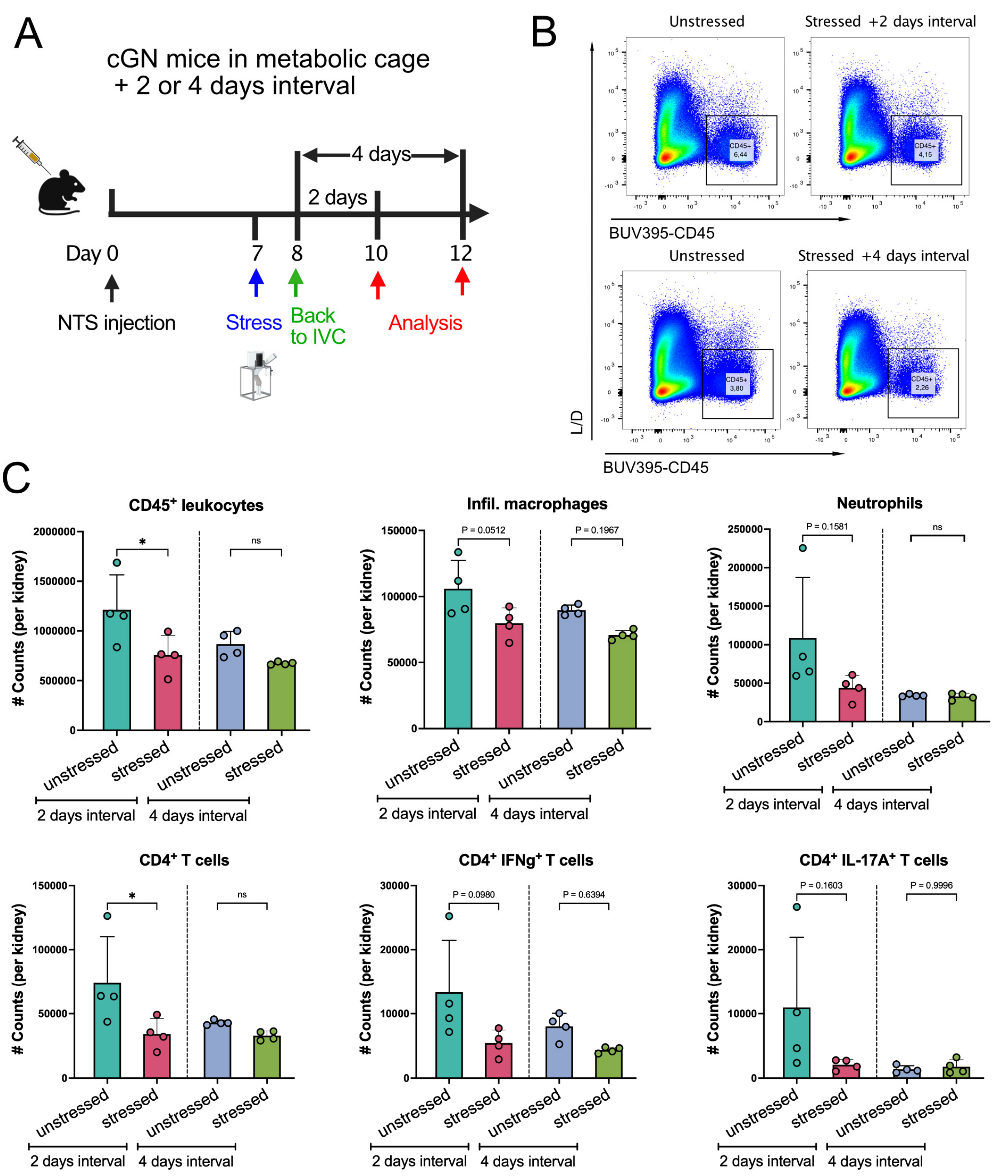

Supplemental Fig. 2. Duration of the reduction of nephritis after residence in metabolic cages. (**A**) Experimental diagram for determining the duration of nephritis suppression. Mice were placed in metabolic cages overnight and put back to the IVC, and analyzed 2 or 4 days later. Unstressed mice were housed in the IVC during the entire experiment until organ collection. (**B**) Representative flow cytometry plot of renal $CD45^+$ leukocytes from cGN mice, cGN mice placed in metabolic cages with 2- or 4-days interval before organ collection. (**C**) Bar plots showing the renal infiltration of $CD45^+$ leukocytes, infiltrating macrophages, neutrophils, $CD4^+$ T cells, Th1 and Th17 cells from cGN mice, cGN mice placed in metabolic cages with 2- or 4-days interval before organ collection (n=4). Data presented as mean ± SD. Statistical analysis was performed by using one-way ANOVA. P, * < 0.05.

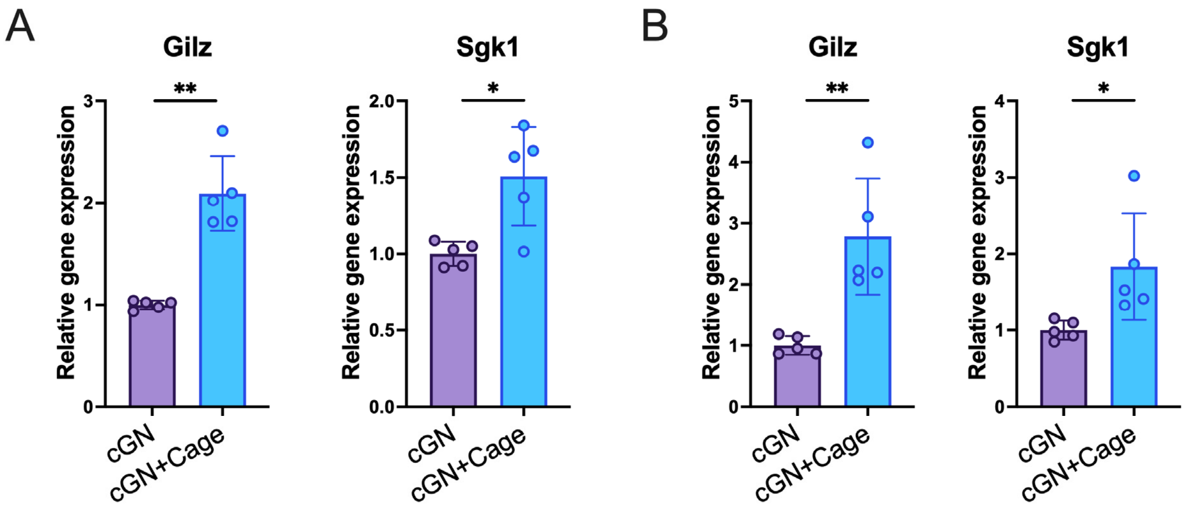

Supplemental Fig. 3. qPCR of glucocorticoids related genes. Relative gene expression of *Gilz* and *Sgk1* from the macrophages (**A**) and CD4$^+$ T cells (**B**) from the kidney of cGN mice placed in metabolic cage or not on day 7. n=5, data were merged from 2 individual experiments. Data presented as mean ± SD. Statistical analysis was performed by using t-test. P, * < 0.05, ** < 0.01.

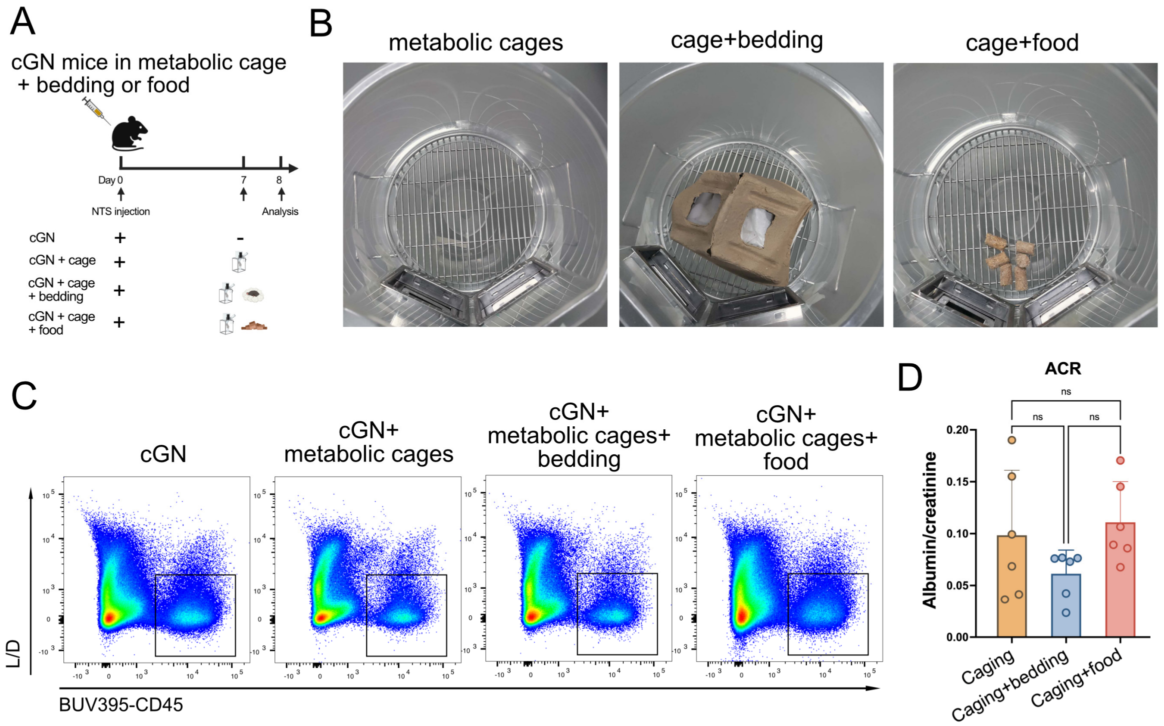

Supplemental Fig. 4. Mice placed in the metabolic cages were treated by adding bedding or food. (**A**) Experimental diagram for determining the effect of adding bedding or food to mice placed in metabolic cages during the progression phase of cGN. (**B**) Micrographs of metabolic cages with or without bedding or food. (**C**) Representative flow cytometry plot of renal CD45+ leukocytes from cGN mice, cGN mice placed in metabolic cages with or without bedding or food. (**D**) Bar plots showing the urine albumin/creatinine ratio (ACR) from cGN mice placed in metabolic cages with or without bedding or food. (n=6, data were merged from 2 individual experiments). Data presented as mean ± SD. Statistical analysis was performed by using one-way ANOVA. P, *** < 0.001, **** < 0.0001.
